# Effect of treatment for early gestational diabetes mellitus on neonatal respiratory distress: A secondary analysis of the TOBOGM study

**DOI:** 10.1111/1471-0528.17938

**Published:** 2024-08-19

**Authors:** David Simmons, Jincy Immanuel, William M. Hague, Suzette Coat, Helena Teede, Christopher J. Nolan, Michael J. Peek, Jeff R. Flack, Mark McLean, Vincent W. Wong, Emily J. Hibbert, Alexandra Kautzky‐Willer, Jürgen Harreiter, Helena Backman, Emily Gianatti, Arianne Sweeting, Viswanathan Mohan, N. Wah Cheung, the TOBOGM Research Group, Raiyomand Dalal, Georgia Soldatos, Suja Padmanabhan, Rohit Rajagopal, Victoria Rudland, Herbert Kiss, Erik Schwarcz, Ranjit Mohan Anjana, Uma Ram

**Affiliations:** ^1^ School of Medicine Western Sydney University Campbelltown New South Wales Australia; ^2^ Robinson Research Institute The University of Adelaide Adelaide South Australia Australia; ^3^ Department of Medicine Monash University Melbourne Victoria Australia; ^4^ Department of Medicine Canberra Hospital and Australian National University Canberra Australian Capital Territory Australia; ^5^ School of Medicine and Psychology Australian National University Canberra Australian Capital Territory Australia; ^6^ Department of Medicine Bankstown‐Lidcombe Hospital Sydney New South Wales Australia; ^7^ Department of Medicine Blacktown Hospital Sydney New South Wales Australia; ^8^ Department of Medicine Liverpool Hospital and University of New South Wales Sydney New South Wales Australia; ^9^ Nepean Clinical School, Faculty of Medicine and Health University of Sydney and Nepean Hospital Sydney New South Wales Australia; ^10^ Gender Medicine Unit, Division of Endocrinology and Metabolism, Department of Medicine III Medical University of Vienna Vienna Austria; ^11^ Department of Medicine Landesklinikum Scheibbs Scheibbs Austria; ^12^ Department of Obstetrics and Gynecology, Faculty of Medicine and Health Örebro University Örebro Sweden; ^13^ Endocrinology and Diabetes Fiona Stanley and Fremantle Hospitals Murdoch Western Australia Australia; ^14^ Department of Endocrinology Royal Prince Alfred Hospital Sydney New South Wales Australia; ^15^ Dr. Mohan's Diabetes Specialities Centre and Madras Diabetes Research Foundation Chennai India; ^16^ Department of Medicine Westmead Hospital Sydney New South Wales Australia

**Keywords:** diagnostic criteria, early gestational diabetes mellitus, first trimester, gestational diabetes mellitus, neonatal intensive care, neonatal respiratory distress, pregnancy, screening

## Abstract

**Objective:**

To identify factors associated with neonatal respiratory distress (NRD) in early Gestational diabetes mellitus (eGDM).

**Design:**

Nested case–control analysis of the TOBOGM trial.

**Setting:**

Seventeen hospitals: Australia, Sweden, Austria and India.

**Population:**

Pregnant women, <20 weeks' gestation, singleton, GDM risk factors.

**Methods:**

Women with GDM risk factors completed an oral glucose tolerance test (OGTT) before 20 weeks: those with eGDM (WHO‐2013 criteria) were randomised to immediate or deferred GDM treatment. Logistic regression compared pregnancies with/without NRD, and in pregnancies with NRD, those with/without high‐dependency nursery admission for ≤24 h with those admitted for >24 h. Comparisons were adjusted for age, pre‐pregnancy body mass index, ethnicity, smoking, primigravity, education and site. Adjusted odds ratios (95% CI) are reported.

**Main Outcome Measures:**

NRD definition: ≥4 h of respiratory support (supplemental oxygen or supported ventilation) postpartum. Respiratory distress syndrome (RDS): Supported ventilation and ≥24 h nursery stay.

**Results:**

Ninety‐nine (12.5%) of 793 infants had NRD; incidence halved (0.50, 0.31–0.79) if GDM treatment was started early. NRD was associated with Caesarean section (2.31, 1.42–3.76), large for gestational age (LGA) (1.83, 1.09–3.08) and shorter gestation (0.95, 0.93–0.97 per day longer). Among NRD infants, >24 h nursery‐stay was associated with higher OGTT 1‐h glucose (1.38, 1.08–1.76 per mmol/L). Fifteen (2.0%) infants had RDS.

**Conclusions:**

Identifying and treating eGDM reduces NRD risk. NRD is more likely with Caesarean section, LGA and shorter gestation. Further studies are needed to understand the mechanisms behind this eGDM complication and any long‐term effects.

**Abbreviations**
aOR: adjusted odds ratio
BMI: body mass index
CI: confidence interval
eGDM: early GDM (<20 weeks' gestation)
GDM: gestational diabetes mellitus
GWG: gestational weight gain
HAPO: hyperglycemia and adverse pregnancy outcomes
LGA: large‐for‐gestational age
lGDM: late GDM (diagnosed >24 weeks' gestation after exclusion of early GDM)
NICU: neonatal intensive care unit
NRD: neonatal respiratory distress
OGTT: oral glucose tolerance test
PGD: pre‐gestational diabetes mellitus
RCT: randomised controlled trial
RDS: respiratory distress syndrome
SCN: special care nursery
SGA: small‐for‐gestational age
TOBOGM: Treatment Of BOoking Gestational diabetes Mellitus
TTN: transient tachypnoea of the newborn
weeks': weeks' gestation
WHO: World Health Organisation

## 1 INTRODUCTION

Neonatal respiratory distress (NRD) occurs in up to 7% of newborns^1^ and can be caused by multiple conditions including transient tachypnoea of the newborn (TTN) and the more severe respiratory distress syndrome (RDS).^2^ Management depends on aetiology and severity, ranging from close observation and supplemental oxygen to supported ventilation. Besides an increased need for nursery admission (to the neonatal intensive care unit [NICU] or the special care nursery [SCN]), both TTN and RDS have long‐term implications for the offspring with an associated 1.7‐fold (95% confidence interval [95% CI] 1.4–2.2) increased risk of being admitted to hospital with a diagnosis of asthma over the following 9 years.^3^

The increased morbidity and mortality from NRD associated with diabetes in pregnancy has been known for over 60 years.^4^, ^5^, ^6^ A recent meta‐analysis reported an odds ratio (OR) of the risk of RDS of 1.57 (95% CI 1.28–1.93) for GDM and 2.66 (95% CI 2.06–3.44) for pre‐gestational diabetes mellitus (PGD: largely type 1 and type 2 diabetes in pregnancy),^7^ with the incidence of RDS among neonates from women with GDM up to 20%.^4^ The risk of TTN is similarly increased by 1.50 (95% CI 1.01–2.41) with GDM.^8^

GDM treatment from 24 to 28 weeks' gestation (weeks') has been shown in randomised controlled trials (RCTs) to reduce the risk of complications, including macrosomia^9^, ^10^ and birth trauma^10^; however, no difference in NRD was found between control and intervention groups.^9^, ^10^ Currently, the only large, randomised trial of early (intervention) versus deferred (control) treatment of GDM diagnosed early in pregnancy (“early GDM” [eGDM]), demonstrated a 43% (95% CI 59–21) reduction in NRD in the intervention group, both as part of a composite outcome and as an independent variable. In addition, a reduction (0.78 days) in nursery stay and a small improvement in Apgar scores were shown.^11^ The aim of this analysis was to compare the characteristics of trial participants (both mothers and neonates) with and without NRD, and then the characteristics of those with NRD who had a nursery stay of ≤24 h with those who had a stay of >24 h, as an index of severity.

## 2 METHODS

### 2.1 Study design/participants

This is a nested case–control analysis of the Treatment of Booking Gestational Diabetes Mellitus (TOBOGM) multicentre RCT of either immediate or deferred treatment of eGDM. TOBOGM used a one‐step, 2‐h 75 g oral glucose tolerance test (OGTT) using World Health Organization (WHO) criteria (fasting glucose ≥5.1 mmol/L, 1‐h glucose ≥10.0 mmol/L, 2‐h glucose ≥8.5 mmol/L), across 17 hospitals in Australia, Sweden, Austria and India, as previously described.^11^, ^12^, ^13^ Women with a fasting glucose ≥6.1 mmol/L and/or 2‐h glucose ≥11.1 mmol/L were excluded from the RCT and treated immediately. Briefly, women aged ≥18 years, with a singleton pregnancy and at least one hyperglycaemia in pregnancy risk factor who provided informed consent were recruited at 4–19^+6^ weeks'. Those with a significant active medical disorder were excluded. Two bands were created from the baseline OGTT for stratification of randomisation and analysis: a lower glucose band (fasting 5.1–5.2 mmol/L and/or 1‐h 10.0–10.5 mmol/L and/or 2‐h glucose 8.5–8.9 mmol/L: equivalent to an OR of 1.75 of adverse pregnancy outcomes using data from the Hyperglycemia and Adverse Pregnancy Outcomes (HAPO) study,^14^ with no higher values), and a more frequent upper glucose band (fasting 5.3–6.0 mmol/L and/or 1‐h ≥10.6 mmol/L and/or 2‐h 9.0–11.0 mmol/L: equivalent to an OR of 2.0 using HAPO data). This study was approved by South‐Western Sydney Local Health District ethics committee. The study design was informed by patient feedback in a pilot study.^13^

### 2.2 Procedures and outcomes

Women with eGDM in the RCT were randomly allocated 1:1 to either immediate treatment or deferred treatment (commenced in those in whom a repeat 2‐h 75 g OGTT at 24–28 weeks' was also diagnostic of GDM), stratified by site and glucose band, by minimisation using an electronic randomiser (Techtonic, UK). Treatment included dietary advice and initiation of glucose monitoring, with pharmacotherapy as necessary using local protocols. Weight, height and birth outcomes were collected from hospital records. Neonates of women within the RCT underwent heel‐prick capillary blood glucose within 1–2 h, and biometry within 72 h, of birth. NRD was treated as per local protocols, including admission to neonatal intensive care (NICU) or special care nursery (SCN). Clinic and study staff and participants were blinded to OGTT results.

Outcomes have previously been defined.^11^, ^12^ NRD was defined as respiratory distress warranting ≥4 h of respiratory support with supplemental oxygen, continuous positive airway pressure, or intermittent positive‐pressure ventilation [or combinations thereof] during the 24 h postpartum. For this paper, a new exploratory RDS variable was defined by ≥4 h of respiratory support with continuous positive airway pressure or intermittent positive‐pressure ventilation and ≥24 h stay in a high‐dependency nursery. Where there was a lack of clarity for this definition from existing data, charts were re‐reviewed (13 participants) with no change in the number of cases with RDS using this definition. Reasons for nursery admission in most cases were multiple; identifying a single reason was generally not possible. Pregnancy hypertension was defined as a composite of pre‐eclampsia, eclampsia and gestational hypertension, excluding women with chronic hypertension. Other outcomes included total gestational weight gain (GWG) from the first clinic visit, Caesarean section, induction of labour, perineal injury, birthweight, large‐for‐gestational age (LGA: >90th centile) and small‐for‐gestational age (SGA: <10th centile) (using ethnicity and sex‐adjusted customised centiles for birthweight [www.gestation.net]), 1–2 h postpartum heel‐prick capillary blood glucose ≤2.2 mmol/L, sum of neonatal skinfolds and neonatal lean body mass and fat mass, which were based on four‐site calliper‐measures using the Catalano equation.^11^, ^12^

### 2.3 Statistical analysis

Analyses were performed in SPSS, version 29.0 (IBM, USA). Those with miscarriage or termination of pregnancy were excluded. Descriptive analyses summarise demographic characteristics. Unadjusted comparisons used analysis of variance for continuous variables and *χ* ^2^ for categorical variables (Fisher's exact test if any cells were <5). Multivariable analyses adjusted for the seven previously^11^ prespecified factors: age and pre‐pregnancy BMI as continuous variables (assuming linearity), current smoking, primigravidity and tertiary qualifications as dichotomised variables, ethnicity assigned to groups (as per Tables 1, 3 and 4) and site/site‐clusters (grouped into four as per the TOBOGM RCT).^11^

**TABLE 1:** Comparison of baseline characteristics between pregnancies complicated by no neonatal respiratory distress (NRD), NRD and either not admitted or admitted to nursery (NICU/SCN) ≤24 h (1 day) or NRD and admitted to NICU/SCN >24 h (1 day). Data show *N* (%) or mean (±95% CI).

Total (*N* = 793)	NRD (all)	No NRD	*p*‐Value	NRD	NRD	*p*‐Value
			NRD (all vs. none)	No or ≤1 day in nursery	>1 day in nursery	≤1 vs. >1 day in nursery
*N* (% total)	99 (12.5%)	694 (87.5%)		35 (4.4%)	64 (8.1%)	
Age (years)	32.7 ± 5.3	32.3 ± 4.8	0.410	33.4 ± 5.5	32.4 ± 5.1	0.377
European (%)	46 (46.5%)	269 (38.8%)	) 0.247	15 (42.9%)	31 (48.4%)	) 0.439
South Asian (%)	21 (21.2%)	214 (30.8%)	)	7 (20.0%)	14 (21.9%)	)
East Asian/South East Asian (%)	12 (12.1)	100 (14.4%)	)	7 (20.0%)	5 (7.8%)	)
Middle Eastern (%)	6 (6.1%)	41 (5.9%)	)	1 (2.9%)	5 (7.8%)	)
Tertiary qualifications (%)	15 (15.2%)	138 (19.9%)	0.264	5 (14.3%)	10 (15.6%)	0.859
Nulliparous (%)	40 (40.4%)	217 (31.3%)	0.069	12 (34.3%)	28 (43.8%)	0.359
Current smoking (%)	11 (11.1%)	34 (4.9%)	0.012	2 (5.7%)	9 (14.1%)	0.319
Family history of diabetes (%)	45 (46.9%)	320 (48.6%)	0.758	13 (37.1%)	32 (52.5%)	0.148
History of PCOS (%)	27 (27.3%)	125 (18.1%)	0.030	7 (20.0%)	20 (31.2%)	0.230
Prior GDM (%)	28 (28.3%)	167 (24.1%)	0.574	9 (25.7%)	19 (29.7%)	0.718
Pre‐pregnancy body mass index (kg/m^2^)	32.8 ± 8.0	31.2 ± 8.1	0.066	32.3 ± 7.5	33.0 ± 8.4	0.686
Gestation at early OGTT (days)	109 ± 16	110 ± 18	0.515	111 ± 16	108 ± 15	0.391
Early FBG (mmol/L)	5.1 ± 0.4	5.1 ± 0.4	0.603	5.1 ± 0.4	5.1 ± 0.5	0.449
Early 1HBG (mmol/L)	9.3 ± 1.9	9.1 ± 2.0	0.420	8.5 ± 2.1	9.6 ± 1.8	0.007
Early 2HBG (mmol/L)	7.4 ± 1.7	7.4 ± 1.6	0.619	7.3 ± 1.8	7.6 ± 1.6	0.415
Number undertaking late OGTT (in the deferred treatment group)	59	295		23	37	
Late FBG (mmol/L)	5.1 ± 0.7	5.0 ± 0.6	0.435	5.1 ± 0.6	5.1 ± 0.8	0.924
Late 1HBG (mmol/L)	9.9 ± 2.2	9.7 ± 2.1	0.437	9.3 ± 2.3	10.3 ± 2.1	0.110
Late 2HBG (mmol/L)	8.0 ± 1.8	7.7 ± 1.8	0.199	7.7 ± 1.7	8.2 ± 1.8	0.305
HbA1c (%) (mmol/mol)	5.3 ± 0.4 (34 ± 4)	5.2 ± 0.3 (33 ± 3)	0.090	5.3 ± 0.3 (34 ± 3)	5.3 ± 0.4 (34 ± 4)	0.970
RCT glucose strata						
Upper glucose band (%)	59 (59.6%)	401 (57.8%)	) 0.732	20 (57.1%)	39 (60.9%)	) 0.713
Lower glucose band (%)	40 (40.4%)	293 (42.2%)	)	15 (42.9%)	25 (39.1%)	)
Trial group						
Early treatment of GDM (%)	37 (37.4%)	363 (52.3%)	) 0.005	12 (34.3%)	25 (39.1%)	) 0.639
Deferred treatment of GDM (%)^a^	45 (45.5%)	193 (27.8%)	)	17 (48.6%)	28 (43.8%)	)
GDM remission^a^	17 (17.8%)	138 (19.9%)	)	6 (17.1%)	11 (17.2%)	)
Insulin treated by the time of birth (%)	56 (56.6%)	315 (45.4%)	0.037	20 (57.1%)	36 (56.3%)	1.0
Metformin treated by the time of birth (%)	19 (19.2%)	108 (15.6%)	0.357	35 (14.3%)	14 (21.9%)	0.432

*Note*: Comparisons across multiple categorical variables have *p* values bracketed. Upper glucose band: fasting glucose 5.3–6.0 mmol/L and/or 1‐h glucose ≥10.6 mmol/L and/or 2‐h glucose 9.0–11.0 mmol/L. Lower glucose band: fasting glucose 5.1–5.2 mmol/L and/or 1‐h glucose 10.0–10.5 mmol/L and/or 2‐h glucose 8.5–8.9 mmol/L with no higher values. All *p* values are 2‐tailed.

Abbreviations: 1HBG, 1‐h blood glucose; 2HBG, 2‐h blood glucose; FBG, fasting blood glucose; GDM, gestational diabetes mellitus; OGTT, 75 g 2‐h oral glucose tolerance test; PCOS, polycystic ovarian syndrome; RCT, randomised controlled trial.

^a^ Control group: Deferred treatment refers to those with GDM confirmed on repeat OGTT while GDM remission refers to those in the deferred treatment group with a normal OGTT at 24–28 weeks' gestation.

For the RCT with the previously unreported exploratory outcome of RDS, the adjusted odds ratio (aOR) and 95% CI of early versus deferred diagnosis were estimated using a binary logistic regression model. There were no missing data.

For the case–control study, forward stepwise binomial logistic regression was used to compare pregnancies with and without NRD. Forward stepwise logistic regression was also used to compare those with NRD either not admitted or with a nursery admission (independent of admission reason) of ≤24 h with those with NRD and a nursery admission (independent of admission reason) of >24 h. It was not possible to disentangle different indications for the nursery admission or length of stay >24 h, but all neonates had documented NRD. The regression was repeated as a binomial logistic regression analysis with block entry as a sensitivity analysis. Only those in both models are commented upon. All tests were two‐sided with *p* < 0.05 determined as significant.

## 3 RESULTS

Of the 793 TOBOGM participants (after exclusion of terminations and miscarriages [*N* = 9]), 99 (12.5%) had pregnancies complicated by NRD. Of all TOBOGM neonates, 64/793 (8.1%) developed NRD and were nursery‐admitted for >24 h and 35/793 (4.4%) developed NRD and either did not need nursery admission or were only admitted for ≤24 h (16/35 [45.7%] and 19/35 [54.3%], respectively). Overall, 15 (2.0%) neonates developed RDS: 8 (2.0%) in the deferred treatment group and 7 (1.8%) in the immediate treatment group (aOR 0.84 [95% CI 0.32–2.54]).

Tables 1 and 2 compare unadjusted characteristics and birth outcomes across the three groups. Age, ethnicity, pre‐pregnancy BMI, history of prior GDM, gestation at early OGTT, HbA1c, proportion within the upper and lower glucose bands, late OGTT glucose concentrations and proportion with treated GDM were similar. However, pregnancies complicated by NRD and nursery admission >24 h were more likely to involve maternal smoking and PCOS, have a shorter gestation (and more preterm birth), have a LGA baby, shoulder dystocia, birth trauma or neonatal hypoglycaemia. Those with NRD and nursery admission >24 h had the highest 1‐h glucose at the baseline early OGTT. Pregnancies without NRD (vs. with NRD of either ≤24 or >24 h duration) were most likely to have been treated early in pregnancy (52.3% vs. 34.3% vs. 39.1% respectively, *p* = 0.019) and least likely to have required Caesarean section (36.4% vs. 60.0% vs. 53.1% respectively, *p* = 0.001). Birthweight and calliper measures were similar across the three groups. In the logistic regression comparing pregnancies associated with and without NRD overall (Table 3), NRD was half (aOR 0.50 [95% CI 0.31–0.79]) as likely with an eGDM diagnosis when GDM treatment was given early. NRD was associated with Caesarean section, LGA and shorter pregnancy duration in both stepwise and multivariable logistic regression. In the logistic regression within pregnancies complicated by NRD (Table 4), nursery admission >24 h was associated with a higher 1‐h glucose on the early OGTT.

**TABLE 2:** Comparison of birth outcomes between pregnancies complicated by neonatal respiratory distress (NRD), No NRD and NRD, either not admitted or admitted to nursery (NICU/SCN) ≤24 h (1 day), or NRD and admitted to nursery >24 h (1 day). Data show *N* (%) or mean (±95% CI).

*N* = 751	NRD (all)	No NRD	*p* Value	NRD	NRD	*p* Value
			NRD (all vs. none)	No or ≤1 day in nursery	>1 day in nursery	≤1 vs. >1 day in nursery
*N* ^a^	99	652		35	64	
Hypertension in pregnancy (%)	17 (17.3%)	61 (9.4%)	0.016	6 (17.1%)	11 (17.5%)	0.968
Maternal weight gain (kg)	6.8 ± 6.1	6.0 ± 6.1	0.316	6.4 ± 4.9	7.1 ± 6.8	0.655
Induction of labour (%)	48 (48.5%)	316 (48.5%)	0.851	16 (45.7%)	32 (51.6%)	0.577
Caesarean section (%)	55 (55.6%)	235 (36.4%)	<0.001	21 (60.0%)	34 (53.1%)	0.510
Emergency Caesarean section	31 (31.3%)	114 (17.6%)	<0.001	6 (17.1%)	25 (39.1%)	0.003
Perineal injury 3rd/4th tear (%)	3 (3.1%)	13 (2.0%)	0.454	1 (2.9%)	2 (3.2%)	1.0
Gestation at birth in days	262 ± 18	269 ± 12	<0.001	267 ± 12	259 ± 20	0.030
Male neonate (%)	59 (59.6%)	327 (50.6%)	0.096	16 (45.7%)	43 (67.2%)	0.037
Stillbirth/death (%)	0 (0%)	5 (0.7%)	1.0	0 (0%)	0 (0%)	
Nursery admission (%)	83 (83.8%)	110 (17.0%)	<0.001	19 (54.3%)	64 (100%)	<0.001
Birth trauma (%)	2 (2.0%)	6 (0.9%)	0.055	0 (0%)	2 (3.1%)	0.330
Shoulder dystocia (%)	8 (8.2%)	14 (2.2%)	0.001	2 (5.7%)	6 (9.7%)	0.707
Birthweight ≥4.5 kg (%)	2 (2.0%)	6 (0.9%)	0.288	1 (2.9%)	1 (1.6%)	1.0
Preterm birth <37/40 weeks' (%)	21 (21.2%)	38 (5.9%)	<0.001	3 (8.6%)	18 (28.1%)	0.038
Early term birth 37–38^+6^ weeks' (%)	37 (37.4%)	280 (42.9%)	0.572	13 (37.1%)	24 (37.5%)	0.972
Need for phototherapy (%)	26 (26.5%)	60 (9.5%)	<0.001	5 (14.7%)	21 (32.8%)	0.059
Heelprick ≤2.2 mmol/L within 1–2 h (%)	24 (28.9%)	94/491 (19.1%)	0.042	10/31 (32.3%)	13/51 (25.5%)	0.308
Heel‐prick glucose <1.6 mmol/L (%)	11 (12.1%)	25/567 (4.4%)	0.003	3/35 (8.6%)	8/56 (14.3%)	0.521
Length (cm)	49.2 ± 3.7	49.8 ± 2.9	0.078	49.7 ± 2.5	48.9 ± 4.1	0.278
Birthweight (kg)	3.235 ± 0.775	3.310 ± 0.540	0.229	3.328 ± 0.642	3.185 ± 0.839	0.384
Birthweight centile (GROW)	58.3 ± 34.1	53.0 ± 30.5	0.114	53.0 ± 32.0	61.2 ± 35.2	0.255
Large for gestational age >90th centile (GROW) (%)	29 (29.3%)	105 (16.2%)	0.002	5 (22.9%)	21 (32.8%)	0.298
Small for gestational age <10th centile (GROW) (%)	11 (11.1%)	69 (10.7%)	0.894	2 (5.7%)	9 (14.1%)	0.206
Sepsis (%)	9 (9.1%)	10 (1.6%)	<0.001	3 (8.6%)	6 (9.4%)	0.969
Number with skinfold calliper measurements	53	441		23	30	
Calculated^b^ fat mass (kg)	0.49 ± 0.22	0.46 ± 0.18	0.262	0.51 ± 0.15	0.48 ± 0.27	0.628
Calculated^b^ lean mass (kg)	2.90 ± 0.41	2.88 ± 0.33	0.750	2.94 ± 0.27	2.86 ± 0.49	0.458
Sum of callipers (mm)	20.8 ± 6.0	20.8 ± 4.9	0.986	21.2 ± 4.2	20.5 ± 7.1	0.677

*Note*: All *p* values are two‐tailed.

Abbreviation: GROW, customised gestation‐related optimal weight data (gestation.net).

^a^ Some % relates to lower *N* due to missing data (all <3%).

^b^ Calculated from skinfold thickness.

**TABLE 3:** Variables included in the final logistic regression model comparing maternal characteristics of pregnancies complicated and not complicated by neonatal respiratory distress (NRD Yes/No).

	*B*	SE	Sig.	Odds ratio	95% Confidence interval	
					Lower	Upper
Early treatment vs. No early treatment	−0.701	0.240	0.003	0.496	0.310	0.794
Current smoker vs. Non‐smoker^a^	0.847	0.424	0.046	2.332	1.016	5.352
Caesarean section vs. No Caesarean section	0.836	0.249	<0.001	2.308	1.418	3.756
Large for gestational age (LGA) vs. Not LGA^a^	0.605	0.266	0.023	1.832	1.088	3.083
Induction vs. Not induced^a^	0.569	0.255	0.026	1.767	1.071	2.914
Gestation at birth (days)	−0.054	0.011	<0.001	0.947	0.927	0.968
Constant	12.052	2.979	<0.001			

*Note*: Forward stepwise logistic regression with the following variables included in the analysis: primigravidity, site‐cluster, tertiary education, current smoker, ethnicity, polycystic ovarian syndrome, treatment group in the RCT, age on entry, body mass index (BMI) at entry, gestation at early oral glucose tolerance test (OGTT), early fasting, 1‐ and 2‐h glucose, HbA1c, maternal height, hypertension in pregnancy, Caesarean section, induction, neonatal sex, large for gestational age (LGA) (>90th centile), small for gestational age (SGA) (<10th centile).

^a^ Not included in the multivariable logistic regression sensitivity analysis.

**TABLE 4:** Variables included in the final logistic regression comparing maternal characteristics of pregnancies complicated by neonatal respiratory distress (NRD) with >24 h stay in the nursery (NICU/SCN) and either No or ≤24 h stay in the nursery (NICU/SCN).

	*B*	SE	Sig.	Odds ratio	95% Confidence interval	
					Lower	Upper
1‐h glucose on early OGTT per mmol/L	0.321	0.124	0.010	1.378	1.080	1.758
Constant	−2.400	1.141				

*Note*: Forward stepwise logistic regression with the following variables included in the analysis: primigravidity, site‐cluster, tertiary education, current smoker, ethnicity, polycystic ovarian syndrome (PCOS), treatment group in the RCT, age on entry, body mass index (BMI) at entry, gestation at early oral glucose tolerance test (OGTT), early fasting^a^, 1‐h and 2‐h glucose, HbA1c, maternal height, hypertension in pregnancy, Caesarean section, induction, neonatal sex, large for gestational age (LGA) (>90th centile), ^a^small for gestational age (SGA) (<10th centile).

^a^ Included in the multivariable logistic regression sensitivity analysis.

## 4 DISCUSSION

### 4.1 Main findings

In this large international, multicentre RCT of treating eGDM, approximately one in eight neonates experienced NRD. This was half as likely with early, compared with late, initiation of GDM treatment. NRD was associated with LGA, while longer nursery stay was associated with a higher 1‐h glucose at the early OGTT, but there was no relationship with maternal BMI. NRD was also associated with the well‐recognised risk factors beyond GDM of Caesarean section and shorter gestational age at birth (including preterm birth).^2^ RDS itself was uncommon, similar between the immediate and deferred treatment groups in the RCT, comparable with other large GDM RCTs at 24–28 weeks',^9^, ^10^ but numbers were otherwise too small for further analysis.

TOBOGM was the first large RCT of treating eGDMand diagnosis of eGDM was associated with high rates of NRD, which were reduced by GDM treatment. As previous RCTs of treatment have not shown a reduction in NRD, and the rates of NRD in TOBOGM were higher than those in previous trials, we postulate three possible, potentially overlapping and interpretations. First, that eGDM and late GDM (lGDM) (i.e., diagnosed after 24 weeks' after exclusion of eGDM) are two different conditions, with eGDM being more similar to type 2 diabetes with longer fetal exposure to hyperglycaemia; second, that the definition of NRD in TOBOGM is more inclusive of the range of NRD (and not just focused on the most severe form [RDS]); and/or third, that, if the same obstetric management were to be implemented, eGDM management will decrease NRD incidence related to hyperglycaemia. Labelling of women as having GDM was not a factor, as similar proportions had eGDM diagnosed in each NRD grouping.

The first two large RCTs of GDM treatment (compared with no treatment) showed no significant difference in RDS (Australian Carbohydrate Intolerance Study in Pregnant Women [ACHOIS]^10^: 5% vs. 4%; Maternal–Fetal Medicine Units Network RCT [MFMUN]^9^: 1.9% vs. 2.9%). More recent trials among those with treated versus untreated hyperglycaemia showed similar use of respiratory support (5.1% vs. 5.6%)^15^ and register‐documented RDS (0.63% vs. 0.48%).^16^ However, compared with the women in these trials of GDM, women with eGDM were more hyperinsulinaemic, insulin‐resistant and obese, and had worse hyperglycaemia and dyslipidaemia.^17^ These GDM RCTs included women with less metabolically disturbed pregnancies, and included women developing GDM later in pregnancy, and so are not comparable to the TOBOGM cohort. In one study of mothers with youth‐onset type 2 diabetes (more metabolically disturbed than the TOBOGM women), 18.6% of babies had respiratory distress requiring surfactant or ventilation.^18^

Beyond the degree of metabolic disturbance, a further likely reason for differences in rates of NRD between these earlier RCTs and TOBOGM is differences in definition. Definitions have ranged from no definition,^9^ “the need for supplemental oxygen in the neonatal nursery beyond four hours after birth”^10^ to “use of respiratory support”.^19^ These definitions probably excluded many of those with clinically important, but less severe, NRD. A retrospective electronic medical record study across the US (*n* = 228 438)^19^ reported incidences of RDS and TTN of 3.0% and 3.4%, respectively, in the background population, 4.0% and 5.1% among those with GDM and 10.0% and 8.3% among those with PGD. Even the background population had higher rates of NRD than the GDM RCTs. Importantly, the increased risk of neonatal morbidity was not attributable to preterm delivery.

Besides being RCTs beyond 24 weeks', these GDM RCTs involved participants with lower BMI, lower fasting and 1‐h glucose than TOBOGM, but a higher 2‐h glucose than TOBOGM. While our multivariable analyses did not find that baseline BMI or glycaemia contributed to the presence of NRD, the 1‐h glucose on the early OGTT was associated with severity of NRD. This may reflect other metabolic disturbances rather than glycaemia per se. Nevertheless, eGDM treatment in TOBOGM, which focused on glucose management, was associated with less NRD.

Past studies have proposed that foetal hyperinsulinaemia is associated with delayed pulmonary maturation^20^ and less surfactant.^21^ The association of NRD with more LGA neonates could also reflect intrauterine foetal exposure to maternal hyperglycaemia, and hence foetal hyperinsulinaemia. However, past GDM RCT secondary analyses demonstrated a substantial reduction in LGA but no reduction in RDS. Those developing GDM after 33 weeks' may also be at higher risk of pregnancy complications, but the impact of treatment on NRD is unclear.^22^ Similarly, preconception preparation may be helpful, but again, insufficient data exist regarding benefits of reducing NRD.^23^

Although in TOBOGM, gestational age at birth was indirectly correlated with risk of NRD as expected, there was no association with the gestation at which the early OGTT and treatment occurred. In the main RCT, the diagnosis and treatment of eGDM had its greatest effect on the composite outcome, which included NRD, when treatment was initiated by 14 weeks'.^11^ However, the lack of association between early OGTT timing and NRD may indicate that this is neither a developmental nor a placental “programming” effect from the first trimester. A possible mechanism may be an influence of hyperglycaemia during the canalicular phase of lung development, which has been suggested to be the most important period that relates to NRD, and which occurs at 16–25 weeks'.^24^

### 4.2 Clinical implications

TOBOGM has demonstrated that early exposure to mild hyperglycaemia may be more important than previously considered. Treatment benefit may not be evident if initiated beyond 24–26 weeks'.^25^ These findings support a review of existing guidelines to include early diagnosis and management of eGDM.

### 4.3 Research implications

The current study only included available clinical variables. Further research is needed to understand the relationship between gestational age, glucose management, maternal hyperinsulinaemia and other metabolic biomarkers and NRD. Long‐term follow‐up of the incidence of asthma and other lung conditions^3^ among the TOBOGM offspring and from other studies of eGDM treatment are also clearly of major importance.

### 4.4 Strengths and limitations

This study has strengths. It is the first study to show that treatment of eGDM is associated with a reduced risk of NRD. It is a nested case–control analysis from a well‐designed and implemented RCT of treatment of eGDM. The participants were from a range of sites and ethnic groups. The data, including a few drop‐outs and missing values, were prospectively and systematically collected using the same methodology. The analyses have excluded women with overt diabetes during pregnancy.

There were limitations. No data on steroid treatment were collected, although there are doubts over their benefits after 37 weeks' among women with GDM and the potential for harm through an increased rate of neonatal hypoglycaemia.^26^ Similarly, no clear diagnosis of what type of NRD, the reason for nursery admission or measures of severity of distress (beyond nursery admission) were documented. Indeed, the reason for nursery admission could be for other causes beyond NRD.

### 4.5 Interpretation

NRD, beyond RDS, is an important complication of eGDM, often associated with neonatal nursery admission and is reduced by early identification and treatment.

## 5 CONCLUSION

Pregnancies complicated by eGDM are associated with significant NRD and this risk is reduced by early treatment. Further studies are needed to understand the mechanisms behind this eGDM complication, and how and why early treatment reduces its incidence. Follow‐up studies are crucial to understanding the association of NRD with other metabolic complications, childhood asthma and other respiratory diseases.

## AUTHOR CONTRIBUTIONS

DS conceived the project, drafted the initial paper draft, was responsible for data curation, and performed the formal analysis. JI assisted with the initial paper draft. All authors contributed to the funding acquisition, investigation, methodology, project administration, writing—review and editing. All authors approved the final version of the manuscript. DS is the guarantor.

## FUNDING INFORMATION

This study is supported by the National Health and Medical Research Council (NHMRC grants 1104231 and 2009326), the Region Örebro Research Committee (grants Dnr OLL‐970566 and OLL‐942177), Medical Scientific Fund of the Mayor of Vienna (project numbers 15205 and 23026), the South Western Sydney Local Health District Academic Unit (grant 2016) and a Western Sydney University Ainsworth Trust Grant (2019). The NHMRC grants underwent national peer review. The funder played no role in conducting the research or writing the paper.

## CONFLICT OF INTEREST STATEMENT

None declared. Neither the funding sources nor the author‐affiliated institutions took part in the trial design, the collection, analysis and interpretation of the data, manuscript writing or the decision to submit it for publication.

## ETHICS APPROVAL

The protocol was approved by local ethics committees in each country commencing with South‐Western Sydney Local Health District Research and Ethics Office, Australia (15/LPOOL/551).

## Data Availability

The data that support the findings of this study are available from the corresponding author upon reasonable request.

## Appendix

The TOBOGM Research Group includes:

David Simmons, Western Sydney University, Campbelltown, NSW, Australia. N. Wah Cheung, Westmead Hospital, Sydney, NSW, Australia. Jincy Immanuel, Western Sydney University, Campbelltown, NSW, Australia; Texas Woman's University, Denton, Texas 76204, USA. William M. Hague, Robinson Research Institute, The University of Adelaide, SA, Australia. Helena Teede, Monash Health and Monash University, Melbourne, VIC, Australia. Christopher J. Nolan, The Australian National University and the Canberra Hospital, Canberra, ACT, Australia. Michael J. Peek, The Australian National University, Canberra, ACT, Australia. Jeff R. Flack, Bankstown‐Lidcombe Hospital, Sydney, NSW, Australia. Mark McLean, Blacktown Hospital, Sydney, NSW, Australia. Vincent Wong, Liverpool Hospital, Sydney, NSW, Australia. Emily Hibbert, Nepean Clinical School, University of Sydney and Nepean Hospital, Sydney, NSW, Australia. Emily Gianatti, Endocrinology and Diabetes, Fiona Stanley and Fremantle Hospitals, Murdoch, WA, Australia. Arianne Sweeting, Department of Endocrinology, Royal Prince Alfred Hospital, Sydney, NSW, Australia. Suzette Coat, Robinson Research Institute, The University of Adelaide, SA, Australia. Raiyomand Dalal, Campbelltown Hospital, Campbelltown, NSW, Australia. Georgia Soldatos, Monash Health and Monash University, VIC, Australia. Suja Padmanabhan, Westmead Hospital, Sydney, NSW, Australia. Rohit Rajagopal, Campbelltown Hospital, Campbelltown, NSW, Australia. Victoria Rudland, Westmead Hospital, Sydney, NSW, Australia. Jürgen Harreiter, Gender Medicine Unit, Division of Endocrinology and Metabolism, Department of Medicine III, Medical University of Vienna, Vienna, Austria. Alexandra Kautzky‐Willer, Gender Medicine Unit, Division of Endocrinology and Metabolism, Department of Medicine III, Medical University of Vienna, Vienna, Austria. Herbert Kiss, Division of Feto‐Maternal Medicine, Department of Obstetrics and Gynecology, Medical University of Vienna, Vienna, Austria. Helena Backman, Department of Obstetrics and Gynecology, Faculty of Medicine and Health, Örebro University, Sweden. Erik Schwarcz, Department of Internal Medicine, Faculty of Medicine and Health, Örebro University, Örebro, Sweden. Viswanathan Mohan, Dr. Mohan's Diabetes Specialities Centre and Madras Diabetes Research Foundation, Chennai, India. Ranjit Mohan Anjana, Dr. Mohan's Diabetes Specialities Centre and Madras Diabetes Research Foundation, Chennai, India. Uma Ram, Seethapathy Clinic & Hospital, Chennai, India.

## References

[1] Kumar A , Bhat BV . Epidemiology of respiratory distress of newborns. Indian J Pediatr. 1996;63:93–98. 10829971 10.1007/BF02823875

[2] Edwards MO , Kotecha SJ , Kotecha S . Respiratory distress of the term newborn infant. Paediatr Respir Rev. 2013;14:29–36. 23347658 10.1016/j.prrv.2012.02.002

[3] Smith GCS , Wood AM , White IR , Pell JP , Cameron AD , Dobbie R . Neonatal respiratory morbidity at term and the risk of childhood asthma. Arch Dis Child. 2004;89:956–960. 15383441 10.1136/adc.2003.045971 PMC1719687

[4] Mortier I , Blanc J , Tosello B , Gire C , Bretelle F , Carcopino X . Is gestational diabetes an independent risk factor of neonatal severe respiratory distress syndrome after 34 weeks of gestation? A prospective study. Arch Gynecol Obstet. 2017;296:1071–1077. 28948345 10.1007/s00404-017-4505-7

[5] Gellis SS , Hsia DYY . The infant of diabetic mother. AMA J Dis Child. 1959;97:1–41. 13605412 10.1001/archpedi.1959.02070010003001

[6] Cordero L , Treuer SH , Landon MB , Gabbe SG . Management of infants of diabetic mothers. Arch Pediatr Adolesc Med. 1998;152:249–254. 9529462 10.1001/archpedi.152.3.249

[7] Li Y , Wang W , Zhang D . Maternal diabetes mellitus and risk of neonatal respiratory distress syndrome: a meta‐analysis. Acta Diabetol. 2019;56:729–740. 30955125 10.1007/s00592-019-01327-4

[8] Fung GP , Chan LM , Ho YC , To WK , Chan HB , Lao TT . Does gestational diabetes mellitus affect respiratory outcome in late‐preterm infants? Early Hum Dev. 2014;90(9):527–530. 10.1016/j.earlhumdev.2014.04.006 24819408

[9] Landon MB , Spong CY , Thom E , Carpenter MW , Ramin SM , Casey B , et al. A multicenter, randomized trial of treatment for mild gestational diabetes. N Engl J Med. 2009;361:1339–1348. 19797280 10.1056/NEJMoa0902430 PMC2804874

[10] Crowther CA , Hiller JE , Moss JR , McPhee AJ , Jeffries WS , Robinson JS . Effect of treatment of gestational diabetes mellitus on pregnancy outcomes. N Engl J Med. 2005;352:2477–2486. 15951574 10.1056/NEJMoa042973

[11] Simmons D , Immanuel J , Hague WM , Teede H , Nolan CJ , Peek MJ , et al. Treatment of gestational diabetes mellitus diagnosed early in pregnancy. N Engl J Med. 2023;388:2134–2144. 10.1056/NEJMoa2214956 37144983

[12] Simmons D , Hague WM , Teede HJ , Cheung NW , Hibbert E , Nolan CJ , et al. Hyperglycaemia in early pregnancy: the treatment of booking gestational diabetes mellitus (TOBOGM) study. A randomized controlled trial. Med J Aust. 2018;209:405–406. 29793404 10.5694/mja17.01129

[13] Simmons D , Nema J , Parton C , Vizza L , Robertson A , Rajagopal R , et al. The treatment of booking gestational diabetes mellitus (TOBOGM) pilot randomised controlled trial. BMC Pregnancy Childbirth. 2018;18:151. 29747594 10.1186/s12884-018-1809-y PMC5946423

[14] The HAPO Study Cooperative Research Group . Hyperglycemia and adverse pregnancy outcomes. N Engl J Med. 2008;358:1991–2002. 18463375 10.1056/NEJMoa0707943

[15] Crowther CA , Samuel D , McCowan LM , Edlin R , Tran T , McKinlay CJ . Lower versus higher glycemic criteria for diagnosis of gestational diabetes. N Engl J Med. 2022;387(7):587–598. 36070709 10.1056/NEJMoa2204091

[16] de Brun M , Magnuson A , Montgomery S , Patil S , Simmons D , Berntorp K , et al. Changing diagnostic criteria for gestational diabetes (CDC4G) in Sweden: a stepped wedge cluster randomised trial. PLoS Med. 2024;21:e1004420. 38976676 10.1371/journal.pmed.1004420 PMC11262657

[17] Harreiter J , Simmons D , Desoye G , Corcoy R , Adelantado JM , Devlieger R , et al. IADPSG and WHO 2013 gestational diabetes mellitus criteria identify obese women with marked insulin resistance in early pregnancy. Diabetes Care. 2016;39:e90–e92. 27208336 10.2337/dc16-0200

[18] TODAY Study Group . Pregnancy outcomes in young women with youth‐onset type 2 diabetes followed in the TODAY study. Diabetes Care. 2021;45:1038–1045. 34880068 10.2337/dc21-1071 PMC9174960

[19] Kawakita T , Bowers K , Hazrati S , Zhang C , Grewal J , Chen Z , et al. Increased neonatal respiratory morbidity associated with gestational and pregestational diabetes: a retrospective study. Am J Perinatol. 2017;34:1160–1168. 28738436 10.1055/s-0037-1604414 PMC6113652

[20] McGillick EV , Morrison JL , McMillen IC , Orgeig S . Intrafetal glucose infusion alters glucocorticoid signaling and reduces surfactant protein mRNA expression in the lung of the late‐gestation sheep fetus. Am J Physiol Regul Integr Comp Physiol. 2014;307:R538–R545. 24990855 10.1152/ajpregu.00053.2014

[21] Dekowski SA , Snyder JM . The combined effects of insulin and cortisol on surfactant protein mRNA levels. Pediatr Res. 1995;38:513–521. 8559602 10.1203/00006450-199510000-00007

[22] Cauldwell M , Chmielewska B , Kaur K , van‐de‐l'Isle Y , Sherry A , Watt Coote I , et al. Screening for lateonset gestational diabetes: are there any clinical benefits? BJOG. 2022;129:2176–2183. 35304972 10.1111/1471-0528.17154

[23] Wu L , Ouyang J , Lai Y , Wu P , Wang Y , Ye Y , et al. Combined healthy lifestyle in early pregnancy and risk of gestational diabetes mellitus: a prospective cohort study. BJOG. 2023;130(13):1611–1619. 37212437 10.1111/1471-0528.17548

[24] Rehman S , Bacha D . Embryology, pulmonary. In: StatPearls [Internet]. Treasure Island: StatPearls Publishing; 2023 [updated 2023 Aug 14]. Available from: https://www.ncbi.nlm.nih.gov/books/NBK544372/ 31335092

[25] Yildiz Atar H , Baatz JE , Ryan RM . Molecular mechanisms of maternal diabetes effects on fetal and neonatal surfactant. Children. 2021;8:281. 33917547 10.3390/children8040281 PMC8067463

[26] Gupta K , Rajagopal R , King F , Simmons D . Complications of antenatal corticosteroids in infants born by early term scheduled caesarean. Diabetes Care. 2020;43:906–908. 31974101 10.2337/dc19-2126

